# Propofol Suppresses LPS-induced BBB Damage by Regulating miR-130a-5p/ZO-1 Axis

**DOI:** 10.1007/s12033-023-00835-7

**Published:** 2023-08-09

**Authors:** Ning Gan, Ying Zhou, Jing Li, Aizhong Wang, Yiyun Cao

**Affiliations:** Department of Anesthesiology, The Sixth People’s Hospital Affiliated to the School of Medicine of Shanghai Jiaotong University, 600 Yishan Road, Shanghai, 200233 China

**Keywords:** Blood-brain barrier, miR-130a-5p, ZO-1, propofol

## Abstract

The blood-brain barrier (BBB) is a highly selective semi-permeable barrier that separates circulating blood from the extracellular fluid of the brain and central nervous system, which is crucial for maintaining brain homeostasis. This study aimed to explore the role of propofol in BBB damage and further evaluate the underlying molecular mechanism. Lipopolysaccharide (LPS) was administered to mice to create an in vivo BBB damage mice model. Additionally, hCMEC/D3 cells as brain microvascular endothelial cells (BMECs) were treated with LPS to establish the in vitro BBB damage cell model. Subsequently, propofol was used for the BBB damage model. Evans blue staining and fluorescein sodium were utilized in the in vivo experiments to demonstrate BBB leakage and BBB permeability. Cell counting kit-8 (CCK-8) assay was used to assess cell viability and the trans-endothelial electrical resistance (TEER) value was measured using an epithelial voltmeter. Furthermore, enzyme-linked immunosorbent assay was performed to measure the levels of the inflammatory cytokines such as interleukin-1β (IL-1β) and tumor necrosis factor-alpha (TNF-α). The levels of miR-130a-5p and zonula occludens-1 (ZO-1) in brain tissues and cells were detected using reverse transcription-quantitative polymerase chain reaction, western blot, or immunofluorescence staining. Furthermore, a dual-luciferase reporter assay was used to demonstrate the association between miR-130a-5p and ZO-1. Propofol treatment suppressed BBB leakage, the amount of fluorescein sodium, and the levels of IL-1β and TNF-α in the LPS-induced BBB damage mice model. Meanwhile, propofol treatment increased the TEER value in the LPS-induced hCMEC/D3 cells. Additionally, propofol treatment significantly down-regulated miR-130a-5p and up-regulated ZO-1. More importantly, miR-130a-5p directly targeted ZO-1 and negatively regulated ZO-1 expression in hCMEC/D3 cells. Furthermore, miR-130a-5p mimic partially reversed the effect of propofol on the TEER value and the levels of inflammatory cytokines such as IL-1β and TNF-α in the LPS-induced hCMEC/D3 cells. Propofol suppressed LPS-induced BBB damage by regulating miR-130a-5p/ZO-1 axis. These findings suggested a potentially effective treatment approach for BBB damage.

## Introduction

The blood-brain barrier (BBB) is a highly selective, semi-permeable barrier that regulates the exchange of substances between the blood and the central nervous system [1]. BBB integrity is significant in maintaining the stability of the brain environment and central nervous system function [2, 3]. The BBB prevents solutes from invading brain tissue. BBB damage, which causes tissue edema and dysfunction, is the underlying cause of numerous central nervous system diseases [3]. Therefore, it is necessary to investigate effective methods to protect BBB in neurological diseases.

The BBB is primarily composed of vascular endothelial cells, perivascular cells, extracellular matrix membranes, and astrocytes [4, 5]. Brain microvascular endothelial cells (BMECs) are important components of the BBB and crucial in maintaining BBB function [4]. Studies have revealed that BMECs express high levels of tight junctions and adhesion connexins, preventing blood substances from infiltrating brain tissues through the intercellular gap [6]. Tight junctions are intercellular permeability barriers that regulate the transmembrane transport of water ions and macromolecules via paracellular pathways [4]. Several studies have demonstrated that changes in tight junction protein (TJP) expression, location distribution, and structural and functional abnormalities may impair the integrity of tight junctions and alter BBB permeability [7, 8]. Zonula occludens-1 (ZO-1), also known as TJP1, is a major component of tight junctions, which indicates its significance as a regulator of paracellular permeability in epithelia and endothelia [8–10].

Propofol is the most commonly [11] utilized intravenous anesthetic in the clinic [12]. Propofol has been reported to have neuroprotective and anti-neuroinflammatory properties that protect neurons in ischemic brain tissue [13, 14]. For instance, propofol protects astrocytes from oxidative stress and promotes astrocyte-mediated neuronal protection [15, 16]. The use of propofol has attracted immense research interests worldwide and emerging evidence suggests that propofol enhances tissue and organ function [11, 17, 18]. To the best of our knowledge, the mechanisms of propofol on BBB damage have not been thoroughly described.

MiRNA is a highly conserved non-coding short-chain RNA with 19–24 nucleotides [19]. By binding to the 3’-UTR regions of the target gene mRNA, miRNA inhibits the translation process and regulates the expression of the target genes [19]. MiRNA is used to regulate and control cell metabolism, proliferation, differentiation, and death. Evidence has suggested that miRNA is crucial for maintaining BBB integrity under inflammatory conditions. miR-130a-5p has been reported to be associated with BBB. A clinical study demonstrated that patients with brain edema following BBB injury had elevated serum levels of miR-130a-5p [20]. Another study also demonstrated that miR-130a-5p impaired BBB function and increased BBB permeability by inhibiting Homeobox A5 expression [21]. However, it is unclear whether propofol attenuated BBB damage by regulating miR-130a-5p.

This study aimed to investigate the exact functions and mechanisms of propofol on BBB damage. The results revealed that propofol suppressed lipopolysaccharide (LPS)-induced BBB damage by regulating the miR-130a-5p/ZO-1 axis, which provided a novel approach for the treatment of BBB damage.

## Materials and Methods

### Animal Studies

The male C57BL/C mice (age, 4–6 weeks) were purchased from Jiangsu Animal Laboratory (Jiangsu, China). The mice were housed under 12 h light/dark cycles at 24–26 ℃ with *ad libitum* access to sterile food and water. Thirty C57BL/6J mice were divided into three groups: the Control group (n = 10), the LPS group (n = 10), and the LPS + Propofol group (n = 10). The mice in the LPS + Propofol group were intravenously administered 20 mg/kg/h propofol for 30 min via the tail vein. Meanwhile, phosphate-buffered solution (PBS, Sigma, USA) was administered to the mice in the control and LPS groups as a negative control. After 7 days of pre-treatment, the mice in the LPS and the LPS + Propofol groups were administered intraperitoneally 5 mg/kg LPS (Sigma, USA) for 12 h before the mice were processed. The mice were then intraperitoneally injected with 200 mg/kg sodium pentobarbital (Sigma, USA), and the serum and brain tissues were collected. This experiment was approved by the Animal Ethics Committee of The Sixth People’s Hospital Affiliated with the School of Medicine of Shanghai Jiaotong University (NO.: 2017-0089).

### Evans Blue Staining

The mice were intravenously injected with 4 mL/kg 2% Evans blue dye at 2 h before euthanasia as previously described [22]. The mice were then infused with heparinized saline through the cardiac ventricle until colorless infusion fluid was obtained from the atrium. The brains were removed and frozen. Frozen slices with a thickness of 10 μm were harvested and observed under an epifluorescence microscope (Leica, Germany).

### Blood-Brain Barrier Permeability Measurement

To assess BBB permeability, the mice were intraperitoneally injected with 200 mg/kg sodium fluorescein 1 h before euthanasia as previously described [23]. The brains were removed and extracted using 30% trichloroacetic acid. The fluorescence intensity was measured using a microplate reader (excitation 440 nm, emission 525 nm, Molecular Devices, USA).

### Enzyme-Linked Immunosorbent Assay (ELISA)

Inflammatory cytokines such as interleukin 1β (IL-1β) and tumor necrosis factor-alpha (TNF-α) in serum, brain tissues, and cells were detected using IL-1β and TNF-α Kits (Nanjing Jiancheng Bioengineering Institute, China) according to the manufacturer’s instruction.

### Cell Culture and Treatment

hCMEC/D3 cells (ATCC, USA) were cultured in Rosewell Park Memorial Institute 1640 medium (Gibco, USA) supplemented with 10% fetal bovine serum (FBS, Gibco, USA), 100 µg/mL penicillin (Sigma, USA), and 100 µg/mL streptomycin (Sigma, USA) in a humidified atmosphere with 5% CO_2_ at 37 ˚C.

### Cell Treatment and Transfection

To mimic the BBB dysfunction model in vitro, hCMEC/D3 cells were treated with 30 µg/mL LPS (Sigma, USA) for 24 h. hCMEC/D3 cells were treated with PBS (Sigma, USA) as the negative control.

For overexpression of miR-130a-5p, the sequences of miR-130a-5p mimic and NC mimic were synthesized by GenePharma (GenePharma, China). The sequences were provided as follows: miR-130a-5p mimic: 5’-GCUCUUUUCACAUUGUGCUACU-3’; NC mimic: 5’-UUUGUACUACACAAAAGUACUG-3’. For in vitro transfection, 100 nM of NC mimic or miR-130a-5p mimic were transfected into hCMEC/D3 cells using Lipofectamine 2000 (Invitrogen, USA) for 24 h according to the manufacturer’s protocols.

### Cell Counting Kit-8 (CCK-8)

hCMEC/D3 cells were cultured in 96-well plates with 1 × 10^4^ cells/well and treated with 20, 40, 80, or 160 µmol/L propofol for 24 h [24–27]. Then, hCMEC/D3 cells were added with 10 µL CCK-8 reagent (Beyotime, China) at 37 ℃ for 1 h. Finally, the optical density was measured at 450 nm using a microplate reader (Bio-Rad, China).

### Trans-Endothelial Electrical Resistance (TEER)

The TEER value was measured using an epithelial voltmeter (World Precision Instruments, USA). Briefly, the medium was removed from the upper chamber, and the cell culture insert was transferred to a new well and washed with PBS. The measurement procedure includes measuring the blank resistance (without cells) and measuring the resistance across the cells. Besides, the values were expressed as Ω*cm^2^. When the values reached 300 Ω*cm^2^, the in vitro BBB model was considered established.

### Dual-Luciferase Reporter Assay

The prediction of 3’-untranslated regions (3’-UTR) of ZO-1 as a binding target of miR-130a-5p was verified by TargetScan. Subsequently, the binding site for miR-130a-5p in the 3’-UTR of ZO-1 was constructed using synthetic oligonucleotides and cloned into pGL3-Firefly-Renilla vector to generate wild-type (Wt)-ZO-1 (5’-UAUAGGAACUUAAAUAAUGUGAA-3’). The mutated 3’-UTR sequence of ZO-1 was cloned into the pGL3-Firefly-Renilla vector to generate mutated (Mut)-ZO-1 (5’-UAUAGGAACUUAAAUCCGCAUAA-3’). The luciferase vectors were constructed by GeneChem Company (Shanghai). hCMEC/D3 cells were co-transfected with luciferase plasmids and miR-130a-5p mimic for 2 h using Lipofectamine 2000. The relative luciferase activities were detected by the dual-luciferase reporter assay (Promega, USA). Renilla signals were used to normalize luciferase activity.

### Immunofluorescence Staining

hCMEC/D3 cells were fixed in 4% paraformaldehyde for 15 min and permeabilized with 0.5% Triton X-100 for 20 min. After blocking with goat serum, the cells were incubated with primary antibody ZO-1 (ab190085, 1:100, Abcam, USA) at 4 ℃ overnight and incubated with FITC-labeled secondary antibody at 37 ℃ for 1 h. Subsequently, the cells were stained with 4’,6-diamidino-2-phenylindole (Beyotime, China). The images were observed using a fluorescence microscope (Nikon, Japan).

### Reverse Transcription-Quantitative Polymerase Chain Reaction (RT-qPCR)

Total RNA from tissues and cells was isolated using TRIzol^®^ reagent (Qiagen, USA), and the RNA concentration was detected by NanoDrop (Thermo, USA). Subsequently, total RNA was reverse-transcribed to cDNA by HiScript® III 1st Strand cDNA Synthesis Kit (Vazyme, China). RT-qPCR was implemented on ABI Real-Time PCR System (Applied Biosystems, USA) using SYBR Green Master Mix and TaqMan MicroRNA Assay Kit (Takara, Japan). The expression levels of all genes were calculated using the 2^−ΔΔCT^ method and were normalized to U6 or glyceraldehyde-3-phosphate dehydrogenase (GAPDH). The primer sequences of all genes were listed as follows: ZO-1, forward, 5’-TCACGCAGTTACGAGCAAGT-3’ and reverse, 5’-TGAAGGTATCAGCGGAGGGA-3’; GAPDH, forward, 5’-GATTTGGTCGTATTGGGCGC-3’ and reverse, 5’-TTCCCGTTCTCAGCCTTGAC-3’; miR-130a-5p, forward, 5’-AACACGCGCTGACTCCTAGT-3’ and reverse, 5’-CAGTGCAGGGTCCGAGGT-3’; U6, forward, 5’-GACAGATTCGGTCTGTGGCAC-3’ and reverse, 5’-GATTACCCGTCGGCCATCGATC-3’.

### Western Blot

The total protein from tissues or cells was extracted with ice-cold radio-immunoprecipitation assay lysis buffer (Beyotime, China) plus phenylmethylsulphonyl fluoride (Beyotime, China), and the protein concentrations were detected by bicinchoninic acid kit (Beyotime, China). Subsequently, the protein samples were separated in 10% sodium dodecyl sulfate-polyacrylamide gel electrophoresis (SDS-PAGE) and transferred into polyvinylidene fluoride membranes. The membranes were incubated with primary antibodies ZO-1 (ab190085, 1:1000, Abcam, USA) or GAPDH (ab9485, 1:2500, Abcam, USA) at 4 ℃ overnight. Afterward, the membranes were incubated with secondary antibodies (Abcam, USA) for 1 h at 25℃. Finally, an enhanced chemiluminescence kit (ThermoFisher, USA) was used to visualize the membranes and the protein bands were analyzed by ImageJ software.

### Statistical Analysis

All experiments were repeated thrice independently. Data were analyzed using the Prism Graphpad 8.0 software and presented as means ± standard deviation. A paired student’s t-test was employed to compare two groups, and a one-way analysis of variance followed by Tukey’s poc host was performed to analyze differences among multiple groups. *P* < 0.05 was considered statistically significant.

## Results

### Propofol Alleviates LPS-induced BBB Damage, Inhibits miR-130a-5p, and Promotes ZO-1 Expression in Vivo

**Fig. 1:**
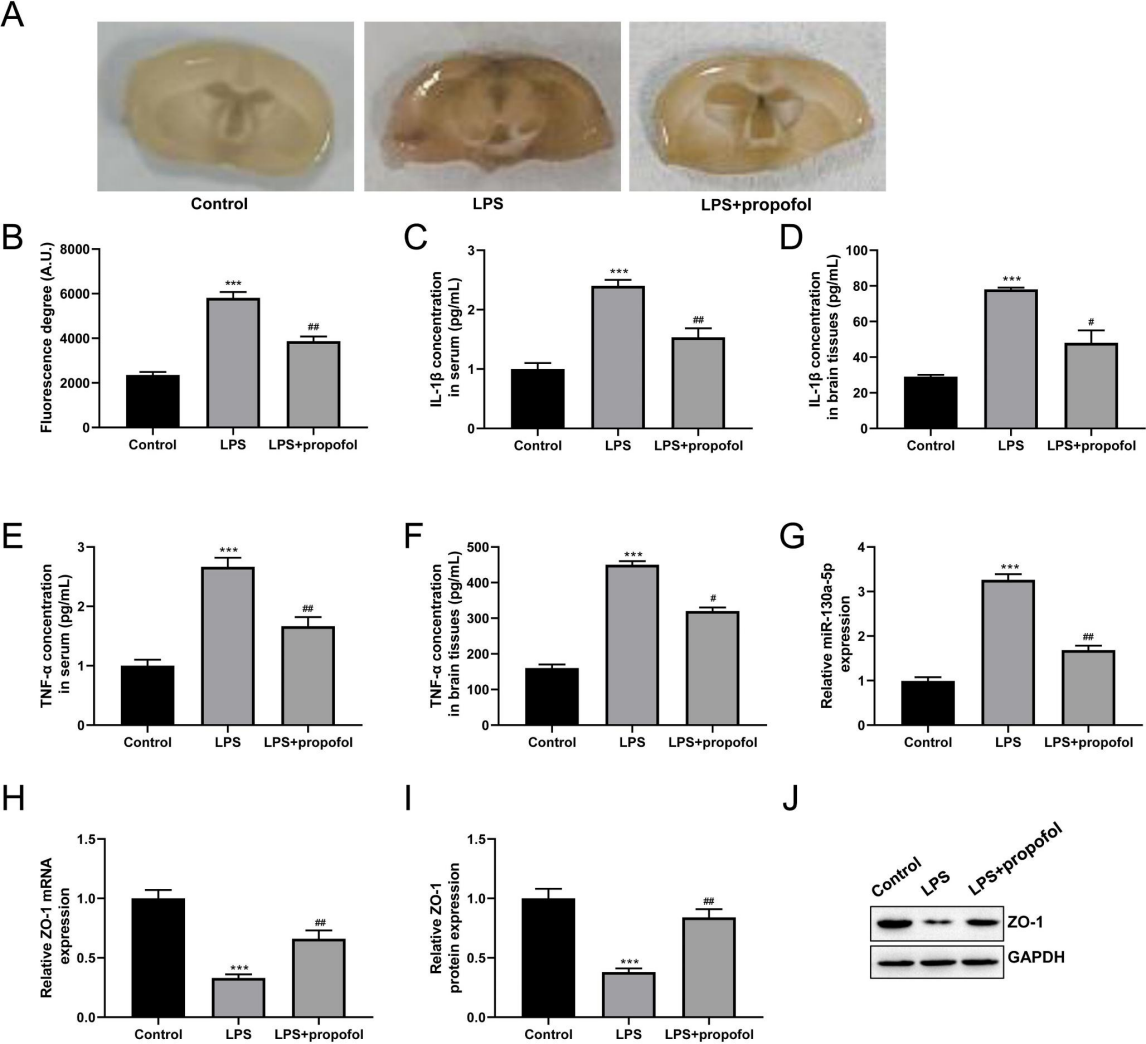
Propofol alleviates lipopolysaccharide (LPS) induced-blood-brain barrier (BBB) damage, inhibits miR-130a-5p, and promotes zonula occludens-1 (ZO-1) expression in vivo. **(A)** Evans blue staining was used to indicate BBB leakage; n = 10. **(B)** The amount of fluorescein sodium in the brain tissue was measured; n = 10. **(C)** Enzyme-linked immunosorbent assay (ELISA) was performed to assess the content of interleukin (IL)-1β in brain tissues; n = 10. **(D)** ELISA was performed to assess the content of tumor necrosis factor-alpha (TNF-α) in brain tissues; n = 10. **(E)** ELISA was performed to assess the content of IL-1β in serum; n = 10. **(F)** ELISA was performed to assess the content of TNF-α in serum; n = 10. **(G)** The expression level of miR-130a-5p in the LPS-induced BBB damage mice model was assessed by reverse transcription-quantitative polymerase chain reaction (RT-qPCR) assay; n = 10. **(H)** The mRNA expression level of ZO-1 in the LPS-induced BBB damage mice model was detected by RT-qPCR assay; n = 10. **(I)** Statistical result of western blot assay; n = 10. **(J)** Western blot assay was performed to analyze the protein expression level of ZO-1 in the LPS-induced BBB damage mice model. ^***^P < 0.001 vs. Control group; ^#^*P* < 0.05, ^##^*P* < 0.01 vs. LPS group

Propofol was injected into the LPS-induced BBB damage mice model to investigate its influence on BBB damage. Evans blue staining revealed that BBB permeability was higher in the LPS group than in the control group; however, it was lower in the LPS group following propofol administration (Fig. 1A). Additionally, LPS increased the amount of fluorescein sodium in the brain tissue, depicting that the BBB was damaged and that the permeability had increased (*P* < 0.001), whereas propofol treatment reversed this effect (Fig. 1B, *P* < 0.01**)**. According to ELISA results, LPS significantly increased the levels of IL-1β and TNF-α in brain tissues and serum (*P* < 0.001), which were further reduced by propofol treatment (Fig. 1C-F, *P* < 0.05**)**. We then determined the expression levels of miR-130a-5p and ZO-1 in the LPS-induced BBB damage mice model. RT-qPCR assay revealed that miR-130a-5p level was significantly increased in the LPS group compared with that in the control group (*P* < 0.01); however, propofol administration dramatically down-regulated miR-130a-5p (Fig. 1G, *P* < 0.01**)**. Furthermore, RT-qPCR and western blot assays demonstrated that LPS dramatically suppressed ZO-1 expression (*P* < 0.001), while propofol treatment reversed this reduction (Fig. 1H-J, *P* < 0.01**)**. These data demonstrated that propofol reduced LPS-induced BBB damage in vivo and controlled the levels of miR-130a-5p and ZO-1.

### Propofol Suppresses LPS-induced BBB Damage, Decreases miR-130a-5p, and Increases ZO-1 Expression in Vitro

**Fig. 2:**
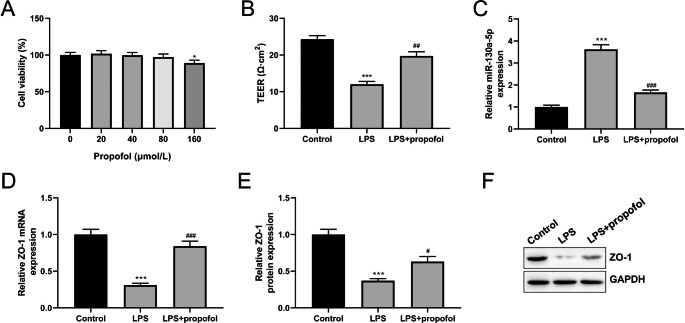
Propofol suppresses lipopolysaccharide (LPS) induced-blood-brain barrier (BBB) damage, decreases miR-130a-5p, and increases zonula occludens-1 (ZO-1) expression in vitro. **(A)** Cell counting kit-8 (CCK-8) assay was used to determine the viability of hCMEC/D3 cells with the different concentrations of propofol (0, 20, 40, 80, and 160 µmol/L) treatment; n = 3. **(B)** The trans-endothelial electrical resistance (TEER) value in hCMEC/D3 cells with LPS and propofol treatment was measured using an epithelial voltmeter; n = 3. **(C)** The expression level of miR-130a-5p in hCMEC/D3 cells with LPS and propofol treatment was assessed using reverse transcription-quantitative polymerase chain reaction (RT-qPCR) assay; n = 3. **(D)** RT-qPCR assay was used to detect ZO-1 mRNA expression in hCMEC/D3 cells with LPS and propofol treatment; n = 3. **(E)** Statistical results of western blot assay; n = 3. **(F)** Western blot assay was performed to analyze the protein expression level of ZO-1 in hCMEC/D3 cells with LPS and propofol treatment. ^*^P < 0.05, ^***^P < 0.001 vs. Control group; ^#^*P* < 0.05, ^##^*P* < 0.01, ^###^*P* < 0.001 vs. LPS group

To further investigate whether propofol modulated BBB damage in vitro, hCMEC/D3 cells were induced by LPS to create an in vitro BBB model. The CCK-8 assay was used to evaluate the viability of hCMEC/D3 cells after propofol treatment at different concentrations (0, 20, 40, 80, and 160 µmol/L). The results demonstrated that when propofol concentrations reached 160 µmol/L, hCMEC/D3 cell viability was significantly decreased (Fig. 2A, *P* < 0.05**)**. Thus, in subsequent experiments, 80 µmol/L of propofol was used to treat hCMEC/D3 cells. Functionally, compared to the control group, LPS dramatically decreased the TEER value (*P* < 0.001); however, compared to the LPS group, propofol treatment increased the TEER value (Fig. 2B, *P* < 0.01**)**. LPS notably increased miR-130a-5p level as determined by RT-qPCR assay (*P* < 0.001), whereas propofol treatment eliminated LPS/s action as a promoter of miR-130a-5p (Fig. 2C, *P* < 0.001**)**. Furthermore, RT-qPCR and western blot assay indicated that the mRNA and protein levels of ZO-1 were decreased in LPS-treated hCMEC/D3 cells (*P* < 0.001), which were reversed by propofol treatment (Fig. 2D-F, *P* < 0.05, *P* < 0.001**)**. Most importantly, propofol treatment blocked the LPS’s ability to promote BBB damage in vitro and regulated miR-130a-5p and ZO-1 levels.

### miR-130a-5p Directly Targets ZO-1

**Fig. 3:**
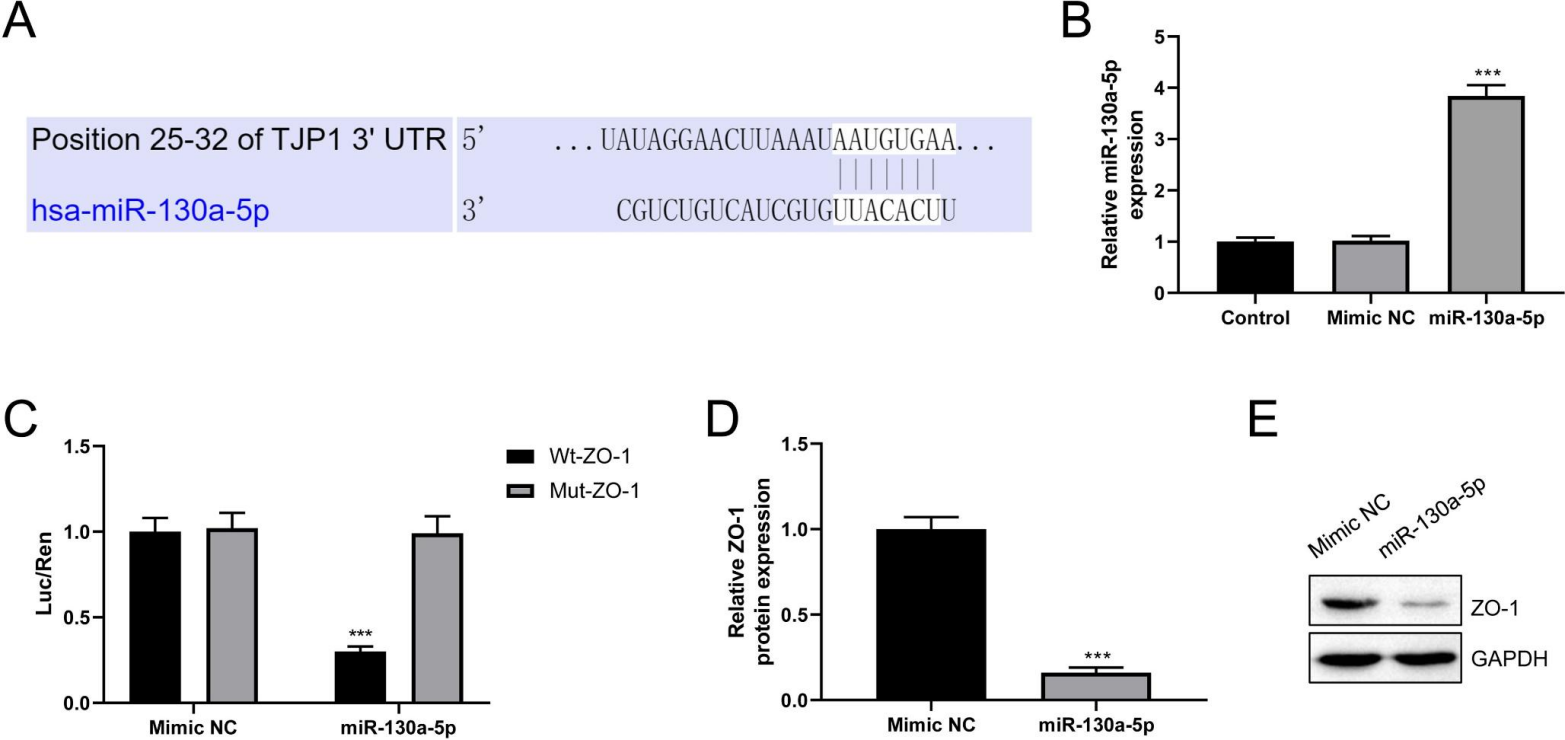
miR-130a-5p directly targets zonula occludens-1 (ZO-1). **(A)** TargetScan identified the potential binding sites between miR-130a-5p and ZO-1. **(B)** The efficiency of miR-130a-5p was confirmed by reverse transcription-quantitative polymerase chain reaction (RT-qPCR) assay; n = 3. **(C)** Dual-luciferase reporter assay was used to assess the relationship between miR-130a-5p and ZO-1; n = 3. **(D)** Statistical results of western blot assay; n = 3. **(E)** Western blot assay was conducted to analyze the protein expression level of ZO-1 in hCMEC/D3 cells with miR-130a-5p mimic transfection. ^***^P < 0.001 vs. Mimic negative control (NC) group

To elucidate the regulatory mechanism of ZO-1 in regulating BBB damage, the online database TargetScan was used to predict the upstream of ZO-1. TargetScan identified the putative binding sites between miR-130a-5p and ZO-1 (Fig. 3A). Afterward, miR-130a-5p mimic was transfected into hCMEC/D3 cells, and the effectiveness of miR-130a-5p was determined by RT-qPCR assay (Fig. 3B, *P* < 0.001**)**. Dual luciferase reporter assay results exhibited that miR-130a-5p mimic significantly decreased the luciferase activity of the Wt-ZO-1 group as opposed to the Mut-ZO-1 group (Fig. 3C, *P* < 0.001**)**. Western blot revealed that miR-130a-5p mimic dramatically suppressed ZO-1 protein level (Fig. 3D-E, *P* < 0.001**)**. ZO-1 expression was overall negatively influenced by miR-130a-5p’s association with it.

### miR-130a-5p Reverses the Protective Role of Propofol in LPS-Induced BBB Damage

**Fig. 4:**
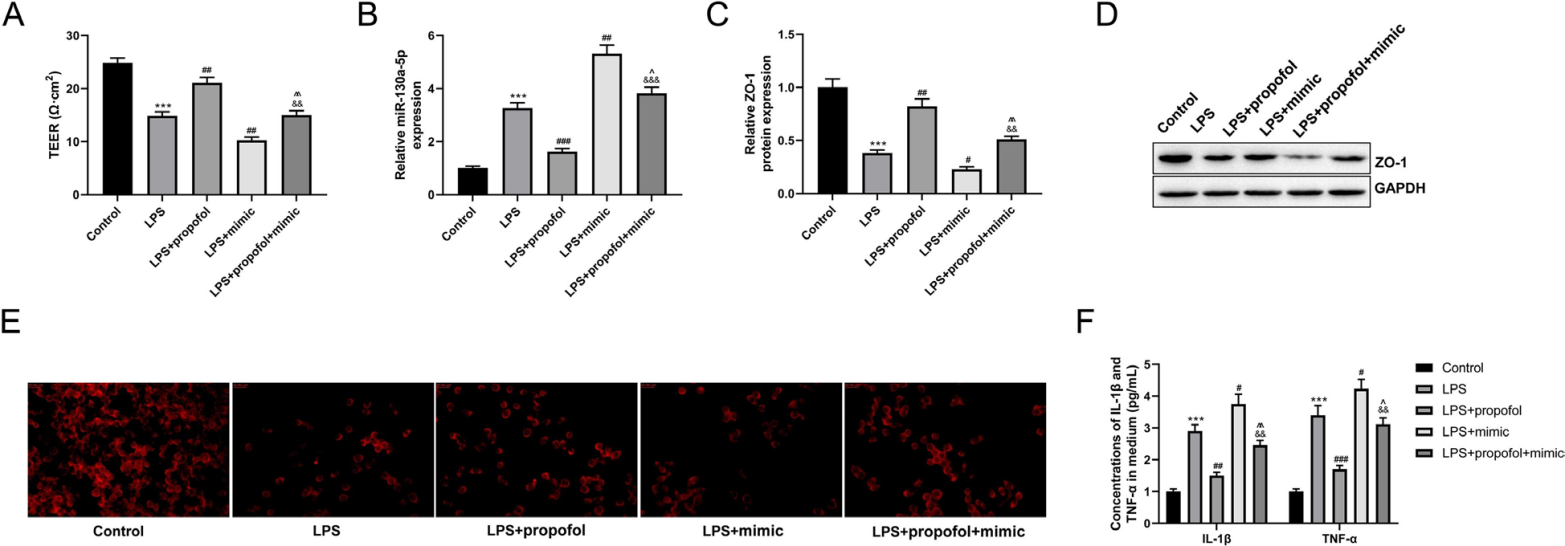
miR-130a-5p reverses the protective role of propofol on lipopolysaccharide (LPS) induced blood-brain barrier (BBB) damage. **(A)** The trans-endothelial electrical resistance (TEER) value in hCMEC/D3 cells with LPS and propofol treatment and miR-130a-5p mimic transfection was measured using an epithelial voltmeter; n = 3. **(B)** The expression level of miR-130a-5p in hCMEC/D3 cells with LPS and propofol treatment and miR-130a-5p mimic transfection was assessed by reverse transcription-quantitative polymerase chain reaction (RT-qPCR) assay; n = 3. **(C)** Statistical results of western blot assay; n = 3. **(D)** The protein level of zonula occludens-1 (ZO-1) in hCMEC/D3 cells was detected by western blot assay. **(E)** Immunofluorescence staining was performed to analyze the protein expression level of ZO-1 in hCMEC/D3 cells; n = 3. **(F)** Enzyme-linked immunosorbent assay (ELISA) was performed to assess the contents of tumor necrosis factor-alpha (TNF-α) and interleukin (IL)-1β in hCMEC/D3 cells. ^***^P < 0.001 vs. Control group; ^#^*P* < 0.01, ^##^*P* < 0.01, ^###^*P* < 0.01 vs. LPS group; ^&&^*P* < 0.01, ^&&&^*P* < 0.001 vs. LPS + propofol group; ^^^*P* < 0.05, ^^^^*P* < 0.01 vs. LPS + mimic group

To determine whether propofol contributed to LPS-induced BBB damage by regulating miR-130a-5p, hCMEC/D3 cells were co-treated with LPS and propofol and transfected with miR-130a-5p mimic. The data validated that propofol treatment increased the TEER value (*P* < 0.01) when compared to the LPS group, whereas miR-130a-5p mimic partially reversed the impact of propofol (Fig. 4A, *P* < 0.01**)**. RT-qPCR assay demonstrated that miR-130a-5p was down-regulated in propofol treated-hCMEC/D3 cells compared to LPS treated-hCMEC/D3 cells (*P* < 0.001); miR-130a-5p mimic dramatically increased miR-130a-5p expression (Fig. 4B, *P* < 0.001**)**. Furthermore, western blot and immunofluorescence staining indicated that the protein level of ZO-1 was increased following propofol treatment (*P* < 0.01) whereas miR-130a-5p mimic inhibited the up-regulation (Fig. 4C-E, *P* < 0.01**)**. Additionally, miR-130a-5p overexpression in hCMEC/D3 cells reversed propofol-induced downregulation of IL-1β and TNF-α as indicated by ELISA (Fig. 4F, *P* < 0.01**)**. All these findings suggested that propofol prevented LPS-induced BBB damage by controlling the expression of miR-130a-5p.

## Discussion

The BBB plays a crucial role in maintaining the physiological state of the central nervous system [1]. Once BBB is destroyed, a substantial amount of material enters the brain parenchyma and BBB permeability changes [28]. BMEC injury, tight junction opening, increased vesicle transport, and astrocyte swelling are the major factors contributing to alterations in BBB permeability [29]. It has been reported that BBB damage is closely associated with several degenerative diseases, such as ischemic stroke, Alzheimer’s disease, and vascular cognitive impairments [30–32]. Therefore, improving our understanding of the potential mechanisms of BBB degradation is crucial for creating better treatment strategies.

LPS, the primary component of Gram-negative bacteria, triggers an inflammatory response that damages neurons [33]. Several studies have reported that LPS causes systemic inflammation and the release of inflammatory cytokines such as IL-1β and TNF-α, which ultimately disrupts the integrity of BBB [33, 34]. Tight junctions are the dynamic structures present between BMECs [4]. The primary structure of the barrier is mainly formed by claudin-1, and occludin and ZO-1 increase its stability [35, 36]. Wei CC et al. demonstrated that alterations in claudin-1, occludin, and ZO-1 expression and distribution altered the stability of tight junctions, resulting in brain injury [37]. Previous studies have revealed that LPS dramatically reduces the levels of TJPs such as occludin and ZO-1 and changes their distribution [38]. ZO-1 is an extremely significant structural protein in the tight junction complex and is the first tight junction adhesive protein that has been verified [39]. To contrast, ZO-1 links to transmembrane proteins. It links with the cytoskeleton protein on the inner side of the cell, forming an essential junction. ZO-1 can be employed as an evaluation indicator of BBB damage and function since its abnormal expression is correlated with the severity of BBB injury [34]. In the study, LPS was used to create an in vivo and in vitro BBB damage model. We discovered that LPS triggered injury in hCMEC/D3 cells and caused BBB damage in C57BL/C mice.

Numerous studies have reported that low-dose propofol exhibits a neuroprotective effect [40, 41]. To exemplify, Zhang T et al. clarified that propofol inhibited the activation of microglia by downregulating Cx43, reduced the contents of inflammatory cytokines IL-1β, IL-6, and TNF-α, and played an anti-I/R injury role [40]. Additionally, Chen W et al. suggested that controlling ZO-1 protected against BBB integrity caused by hypoxia [41]. In this study, propofol intervention dramatically reduced BBB leakage, suppressed inflammatory response, and preserved the structural integrity of BBB. Moreover, propofol significantly reduced BBB damage to brain tissue in mice and BMECs. The underlying mechanisms may be associated with the down-regulation of miR-130a-5p and up-regulation of ZO-1.

Numerous studies have demonstrated that miRNA contributes to the preservation of BBB integrity [42, 43]. To illustrate, it has been noted that the inhibition of the miR-132/212 cluster increases infarct volume and damages BBB [42]. Wu J et al. hypothesized that miR-9-5p ameliorated the BBB damage and neuroinflammatory response caused by oxygen-glucose deprivation (OGD) by targeting Ptch-1 [43]. Currently, the bioinformatics prediction approach of miRNA target genes relies on the principle of base matching between the miRNA sequence and the corresponding target mRNA sequence. In this study, TargetScan and dual-luciferase reporter assay validated the direct targeting of ZO-1 by miR-130a-5p. Recently, some studies have demonstrated that miR-130a-5p has multiple biological functions, such as regulating cell behaviors including cell growth, apoptosis, and angiogenesis [44, 45]. Sui S et al. revealed that miR-130a-5p promoted cell viability and angiogenesis in OGD induced-BMECs [44]. In this study, miR-130a-5p reversed the protective role of propofol on LPS-induced BBB injury.

## Conclusion

Propofol exhibits a significant protective impact on LPS-induced BBB damage. The mechanism of propofol includes decreased miR-130a-5p and increased ZO-1, thus, reducing the release of inflammatory cytokines and maintaining the integrity of BBB.

## Data Availability

The datasets used or analyzed during the current study are available from the corresponding author on reasonable request.
